# MicroRNA-126 overexpression rescues diabetes-induced impairment in efferocytosis of apoptotic cardiomyocytes

**DOI:** 10.1038/srep36207

**Published:** 2016-11-09

**Authors:** Sahana Suresh Babu, Rajarajan A. Thandavarayan, Darukeshwara Joladarashi, Prince Jeyabal, Shashirekha Krishnamurthy, Arvind Bhimaraj, Keith A. Youker, Prasanna Krishnamurthy

**Affiliations:** 1 Department of Cardiovascular Sciences, Centre for Cardiovascular Regeneration, Houston Methodist Research Institute, Houston, TX 77030, USA; 2 Department of Biomedical Engineering, Schools of Medicine and Engineering, University of Alabama at Birmingham, AL 35294, USA; 3 Houston Methodist DeBakey Heart & Vascular Center, Houston Methodist Hospital, Houston, Texas, USA

## Abstract

Efferocytosis, a process of clearance of apoptotic cells by phagocytes, is essential for successful resolution of inflammation and maintenance of tissue homeostasis. Diabetes compromises the function of macrophages leading to adverse inflammatory response during wound healing, myocardial injury, atherosclerosis and autoimmune disorders. However, the effect of diabetes on macrophage-mediated efferocytosis of apoptotic cardiomyocytes (ACM) and the molecular mechanisms involved are not understood so far. In the present study we found that invitro efferocytosis of ACM was impaired in macrophages from db/db (diabetic) mice. Macrophages exposed to high glucose (HG) decreases microRNA-126 (miR-126) expression with a corresponding increase in ADAM9 expression. Dual-luciferase reporter assay confirms that ADAM9 3′UTR contains miR-126 target site. ADAM9 inhibition reduces HG-induced proteolytic cleavage of Mer tyrosine receptor kinase (MerTK, a proto-oncogene that plays a critical role in phagocytosis), resulting in shedding of soluble-Mer (sMER) and loss of MERTK function. Over-expression of miR-126 attenuates HG-induced impairment of efferocytosis. Furthermore, human diabetic hearts show lower miR-126 expression with a corresponding increase in ADAM9 expression vs. normal counterparts. These data suggests that diabetes impairs efferocytosis of ACM and that strategies to enhance efferocytosis might attenuate diabetes-induced impairment in inflammation resolution and cardiac repair after injury.

Efferocytosis (phagocytosis or clearance of the apoptotic cells) is a crucial cellular process required for maintaining tissue homeostasis and immune defense^1^. Following tissue injury, resident or infiltrating phagocytes (predominantly macrophages) engulf and clear the dead and apoptotic cells leading to efficient resolution of inflammation and subsequent wound healing process^2^. Diabetes is known to compromise the function of macrophages and is associated with prolonged inflammatory response and adverse cardiac remodeling changes therefore increase the risk for heart failure and alter the disease outcome^3^,^4^. However, the effect of diabetes on macrophage-mediated efferocytosis of apoptotic cardiomyocytes and the molecular mechanisms involved has not been understood so far. Although some of the engulfment ligands involved in efferocytosis has been well studied, the contributions of these ligands in cardiomyocyte efferocytosis under diabetic condition remains to be explored.

microRNAs (miR) have been shown to regulate a range of biological processes during development and disease, including inflammation and wound healing^5^. However, the role of miRs in apoptotic cardiomyocyte efferocytosis under diabetic condition is understudied. Our work presented here demonstrates that miR-126 modulates macrophage-mediated phagocytosis of myocytes under hyperglycemic conditions. miR-126 is a key regulator of angiogenesis and maintains vascular homeostasis and is known to be down regulated during diabetic complications^6^. MiR-126 targets several cytokines, transmembrane proteins involved in a wide range of cellular processes. For instance, miR-126 regulates expression of Human A Disintegrin And Metalloproteases (ADAMs), thus aiding tumor suppression^7^. ADAMs are membrane-anchored proteins that belong to the zinc protease superfamily and has been implicated in cytokine and growth factor shedding and cell migration, as well as pathological processes such as inflammation and cancer^8^. ADAMs cleave several inflammatory cytokines, transmembrane and extra-cellular proteins in a variety of diseases including Alzheimer’s disease, arthritis, and cancer^9^,^10^. In addition, ADAMs cleave Mer tyrosine kinase (MERTK), a member of the TAM (TYRO3, AXL, and MERTK) receptor tyrosine kinases^11^. Proteolytic cleavage of MERTK leads to generation of soluble MERTK (sMER) and reduces intracellular downstream signaling that affects efferocytosis and inflammation resolution *in vivo*^12^.

In the present study, we demonstrate that diabetes-mediated decrease in miR-126 leads to a corresponding increase in its target, ADAM9, which in turn cleaves MERTK (inactivates downstream engulfment signaling), thus resulting in defective macrophage efferocytosis of apoptotic cardiomyocyte. Furthermore, our study also demonstrates the possibility that overexpression of miR-126 could rescue deficient efferocytosis under diabetic condition and therefore might promote efficient resolution of inflammation and wound healing following tissue injury.

## Results

### Diabetic conditions impair efferocytosis of apoptotic cardiomyocytes by macrophages

**Figure 1:**
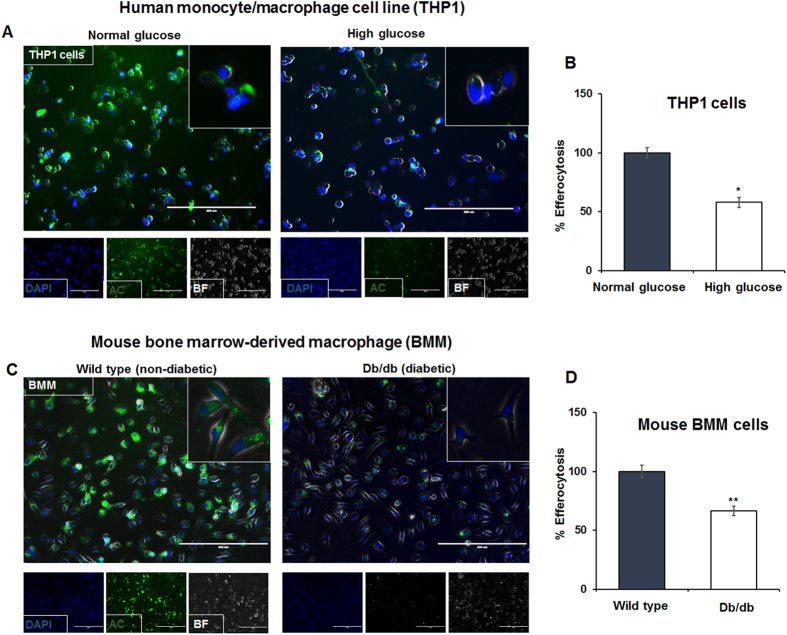
Reduced efferocytosis of apoptotic cardiomyocytes in diabetic conditions. (**A**) Fluorescent microscopy showing engulfment of apoptotic cells (AC; human ventricular cardiomyocytes, SV40) by THP1 cells (human monocyte cell line exposed to high glucose (35 mM glucose). (**B**) Quantitation of efferocytosis showing reduced efferocytosis by THP-1 cells in high glucose condition as compared to cells treated with normal glucose. n = 3, *P < 0.001. (**C**) Fluorescent microscopy showing engulfment of apoptotic cells (AC; human ventricular cardiomyocytes, SV40) by mouse bone marrow derived macrophages (BMM) from non-diabetic and diabetic (db/db) mice. (**D**) Quantitation of efferocytosis showing reduced efferocytosis by macrophages derived from diabetic (db/db) mice compared to cells from wild type (non-diabetic) mice. n = 3, **P < 0.001. Apoptotic cells are labeled with calcein (green) and macrophage nuclei stained with DAPI (blue). At least 100 macrophages were counted and the data represent three independent experiments done in triplicate.

Previous study has shown that macrophage dysfunction impairs resolution of inflammation in cutaneous wounds of diabetic mice^13^. To determine the influence of diabetes on macrophage-mediated efferocytosis, we evaluated engulfment of apoptotic human ventricular cardiomyocytes (SV40 cells, calcein-labeled, green) by THP1 cells (human monocyte cell line) treated with high glucose (HG, 35 mM) or normoglycemia (NG, 5 mM) conditions. THP1 cells exposed to high glucose (HG, 35 mM) showed lower ability to ingest apoptotic cells as compared to macrophages cultured in normoglycemia (NG, 5 mM) conditions (Fig. 1A,B; *P < 0.001). Furthermore, bone marrow-derived macrophages from diabetic (db/db) mice show reduced uptake of apoptotic cardiomyocytes as compared to macrophages from non-diabetic control (wild type) mice (Fig. 1C,D; **P < 0.001). Also, RAW 264.7 cells (mouse macrophage cell line) grown in high glucose (HG, 35 mM) condition shows lower ability to engulf apoptotic cells (data not shown). Interestingly, live cardiomyocytes were not ingested (Supplementary Fig. S1A), suggesting that apoptotic cells but not live cells express certain “eat-me-signals”. Furthermore, performing efferocytosis assay at 4 °C instead of 37 °C (Supplementary Fig. S1B) or pre-incubation of macrophages with *cytochalasin D* (2 μM) to disrupt actin polymerization (Supplementary Fig. S2A) inhibits efferocytosis and did not show engulfment of the apoptotic cells indicating that our assay shows energy-dependent engulfment and not a passive adsorption of dead cells to phagocytes. In addition, flow cytometry analysis (Supplementary Fig. S2B) shows that upon engulfment of pHrodo green dye-labeled apoptotic cells, macrophages exhibit increasing fluorescence (green) due to acidification of endocytic compartments in comparison to the dye being nonfluorescent at neutral pH (or lack of efferocytosis of live cells). Also, representative z-stack image from 3D confocal imaging depicts ingestion of calcein labeled AC (green) by macrophages (Supplementary Fig. S2C). These observations confirm the engulfment of dead cardiomyocytes by macrophages.

### miR-126 deficiency impairs efferocytosis via regulation of ADAM9

Previous reports have shown that miR-126 is a critical regulator of vascular integrity and angiogenesis^14^,^15^ and reduced miR-126 expression during diabetes is predicted as a potential biomarker of type 2 diabetes^16^,^17^,^18^. However, its role in efferocytosis under diabetic conditions is not known. Recent study has demonstrated the essential role of MERTK and potential MERTK cleavage in cardiomyocyte efferocytosis. Although, MERTK is not a direct target of miR-126, computational analyses show that MMP9 and ADAM9 (metalloproteinase involved in proteolytic cleavage) are potential targets^7^,^19^,^20^,^21^.

**Figure 2:**
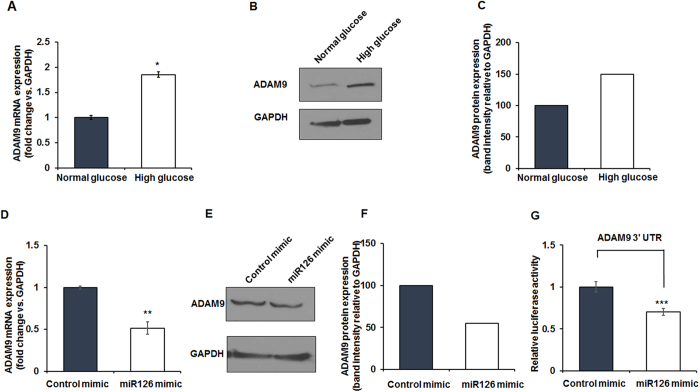
High glucose upregulates ADAM9 expression while MiR-126 mimic inhibits its expression in RAW 264.7 cells. (**A**) Quantitative real time PCR data showing increase in ADAM9 mRNA expression in RAW 264.7 cells treated with high glucose compared to normoglycemia (normal glucose) treated cells. n = 3. *P < 0.01. (**B**) Representative western blot and densitometry (**C**) showing a corresponding increase in ADAM9 protein expression in high glucose-treated cells. (**D**) Reduced ADAM9 mRNA levels in RAW 264.7 cells transfected with miR-126 mimic compared to control mimic treated cells; n = 3, **P < 0.05. (**E**) Representative western blot and densitometry (**F**) showing ADAM9 protein expression decreases in miR-126 mimic treated RAW 264.7 cells compared to controls. (**G**) Quantitative dual-luciferase reporter assay showing decrease in ADAM9 3′UTR-mediated luciferase activity in miR126 mimic treated cells when compared to control mimic treated cells; n = 3. ***P < 0.01. Data normalized to GAPDH.

To determine the effect of diabetes on ADAM-9, we analyzed ADAM9 mRNA and protein expression in macrophages after HG treatment for 48 hours (Fig. 2). Exposure to HG significantly upregulates ADAM9 mRNA (Fig. 2A, *P < 0.01) and protein expression as compared to NG (Fig. 2B,C and Supplementary Fig. S3A). Furthermore, miR-126 expression was lower in HG treated cells as compared to NG treated macrophages (Fig. S3B). To examine the impact of miR-126 on ADAM9 expression, we transfected macrophages with miR-126-specific or control mimics. The effect of miRNA mimic transfection on miR-126 expression was confirmed by RT-PCR (Supplementary Fig. S3C). We observed that miR-126 mimic transfection significantly decreases ADAM9 mRNA (Fig. 2D, **P < 0.05) and protein expression (Fig. 2E,F) as compared to control mimic transfected macrophages.

Next, to validate whether 3′UTR of ADAM9 contains a target sequence for miR-126 binding, macrophages were transfected with a Dual-Luciferase reporter vector containing the 3′-UTR of ADAM9 along with miR-126 mimic or non-specific control mimic. Interestingly, cells transfected with miR-126 mimic showed 30% decrease in luciferase activity (Fig. 2G, ***P < 0.01). Furthermore, we tested if miR-126 overexpression inhibits HG-induced increase in ADAM9 expression. miR-126 mimic transfection decreases HG-induced ADAM9 expression, when compared to control mimic treated macrophages (Supplementary Fig. S3D, *P < 0.05).

### Inhibition of ADAM9 reduces proteolytic cleavage of MERTK

**Figure 3:**
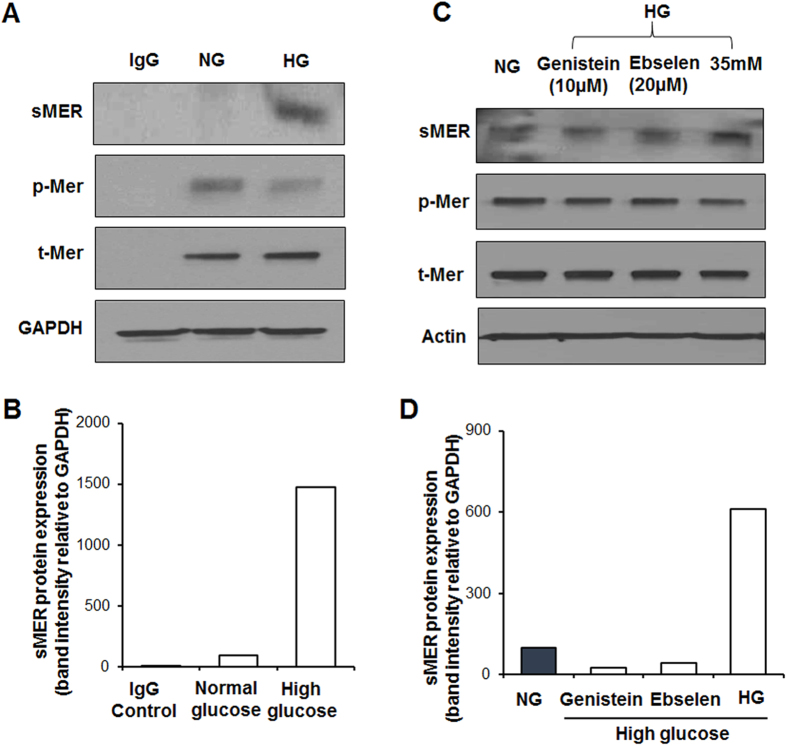
MerTK expression in RAW 264.7 cells exposed to high glucose. (**A**) Immunoprecipitation and western blotting showing higher sMer (soluble form of MERTK) levels in the conditioned media of cells exposed to high glucose (HG) for 48 hrs compared to normoglycemia condition (NG). The corresponding cell lysate showing decrease in phosphorylated MerTK (p-Mer) levels in high glucose treated cells, while total MerTK (t-Mer) was unchanged. IgG antibody used as a negative control. (**B**) Densitometry of sMer in the above blot. (**C**) RAW 264.7 cells pretreated with ADAM blockers, genistein and ebselen, inhibit HG-induced increase in sMer and decrease in p-Mer. (**D**) Densitometry of sMer in the above blot, normalized to GAPDH expression.

Previous studies have shown that proteolysis of an ectodomain fragment of MERTK, known as soluble MER (sMER) inhibits efferocytosis^12^,^22^,^23^. Also, autophosphorylation of Mertk (site Tyr-867) governs phagocytosis and downstream cytoskeletal signaling^24^. Since we observed an increase in ADAM9 expression in macrophages treated with high glucose, the conditioned media from NG and HG treated macrophages was collected and analyzed for inactive soluble Mer (sMer) using immunoprecipitation and Western blotting. Simultaneously, the cell lysates were collected and analyzed for phospho-MerTK (p-Mer). High glucose treatment did not significantly change cellular MERTK expression (Fig. 3A, tMer). Interestingly, sMer levels increased in the condition media of cells treated with HG as compared to NG treated cells (Fig. 3A,B). Furthermore, in association with increased sMER, there is a corresponding decrease in phosphorylation of MerTK (p-Mer) was significantly lower in HG conditions (Fig. 3A).

To test if abrogation of ADAM9 activity rescues high glucose-induced MERTK cleavage, we treated macrophages with small molecules inhibitors of ADAM9 (genistein and ebselen, suppress ADAM9 mRNA and protein, respectively)^25^,^26^ and evaluated sMER and p-MerTK levels. As shown in Fig. 3C,D, inhibition of ADAM9 in HG-stimulated macrophages reduces MERTK shedding and increases phosphorylation.

### Reduced efferocytosis-related signaling in human diabetic hearts

**Figure 4:**
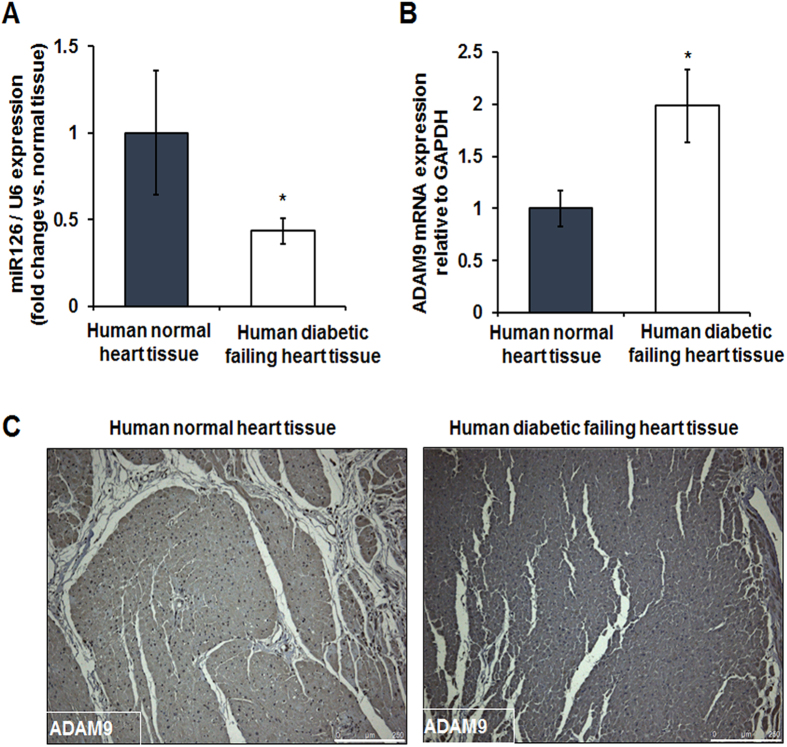
Downregulation of miR-126 and corresponding increase in ADAM9 expression in diabetic human failing heart tissue. (**A**) Quantitative RT-PCR showing lower miR-126 expression in diabetic human failing heart tissue compared to normal heart tissue (normalized to U6, n = 5, *P < 0.05). (**B**) A corresponding increase in ADAM9 mRNA in diabetic failing heart tissues (normalized to GAPDH, n = 3, *P < 0.05). (**C**) Representative immunohistochemistry (IHC) images showing increase in ADAM9 expression in human diabetic failing heart tissue compared to normal human heart tissue.

**Figure 5:**
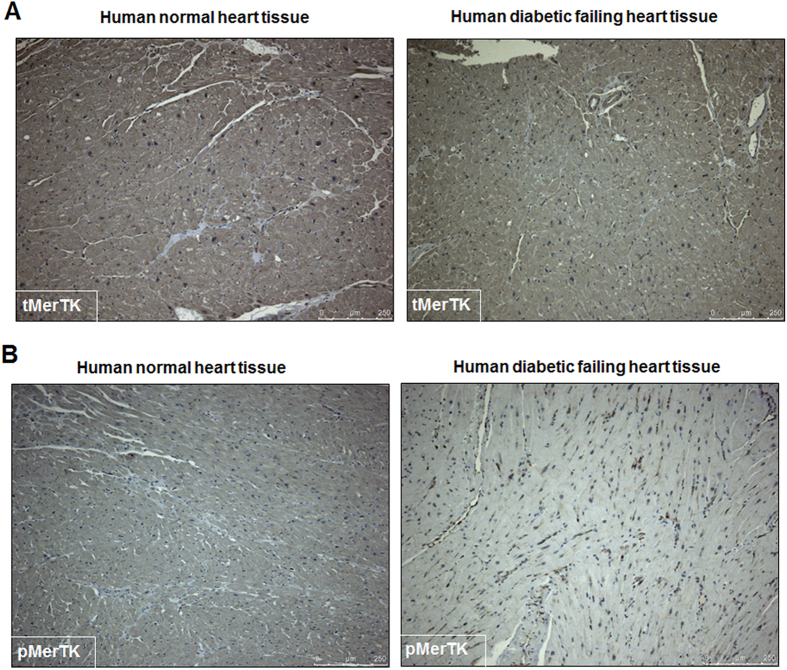
Reduced p-MerTK expression in human diabetic failing heart tissue. Immunohistochemistry image of human heart tissue showing no change in t-MERTK (**A**) and decrease in p-MerTK (**B**) in human diabetic failing heart tissue compared to normal human heart tissue.

To further understand the translational relevance of our findings, we determined the effect of diabetes on miR-126 expression in human failing heart. Cardiac biopsies were collected from left ventricular free wall of failing heart from diabetic patients at the Houston Methodist DeBakey Heart and Vascular Center, Houston Methodist Hospital. Interestingly, miR-126 expression was significantly down-regulated (Fig. 4A, *P < 0.05) in diabetic hearts as compared to non-diabetic heart tissue collected from patients with non-failing heart condition. Consistently, mRNA analysis and histological assessment of the heart tissue samples displayed an increased ADAM9 expression in the diabetic hearts (Fig. 4B,C, *P < 0.05). ADAMs are shown to cleave MerTK leading to generation of soluble form of Mer, which can inhibit efferocytosis both *in-vitro* and *in-vivo*^12^. We analyzed the human tissue samples for the presence of MerTK and its phosphorylation form (pMerTK). Consistent with our *in vitro* data, expression of MerTK did not differ in diabetic and non-diabetic tissues (Fig. 5A; immunohistochemistry). However, pMERTK expression was decreased in the human diabetic failing heart tissue compared to the normal heart tissues (Fig. 5B). In addition, mRNA expression of MerTK did not differ *in-vitro* at 24 hrs after incubation in HG and NG conditions (Supplementary Fig. S4). However, a time course study shows a robust increase in MerTK at 6 & 12 hrs followed by a relative decrease at 48 & 72 hour (Supplementary Fig. S4).

### miR-126 mimics rescue diabetes-induced impairment of efferocytosis

**Figure 6:**
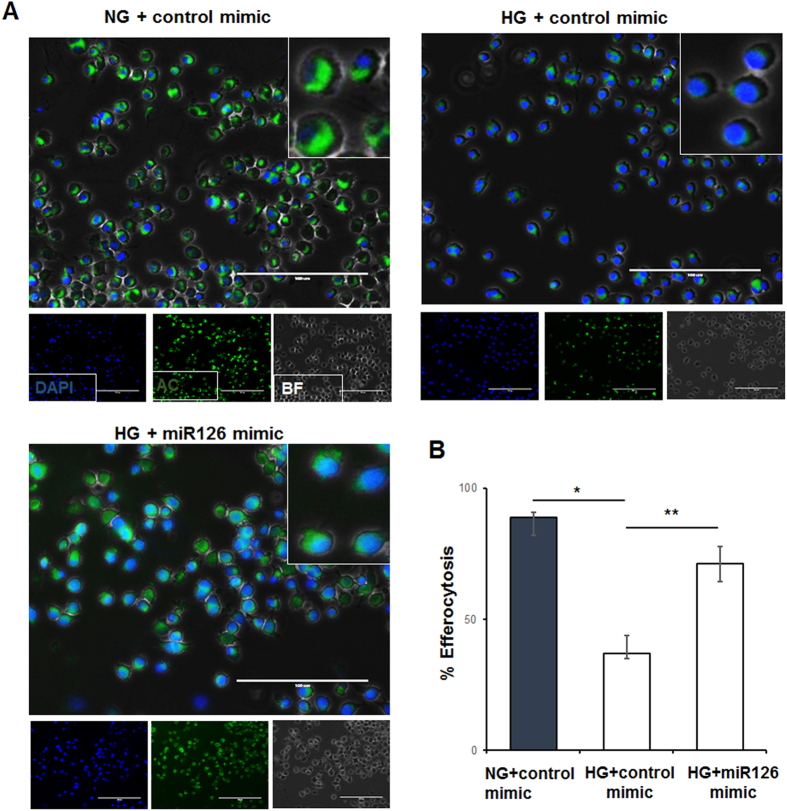
miR-126 mimics restore high glucose-induced impairment of efferocytosis in RAW 264.7 cells. (**A**) Representative fluorescent microscopy images showing reduced macrophage engulfment of apoptotic cells (green) in high glucose treated macrophages. miR-126 mimic transfected macrophages rescue high glucose-induced impairment in efferocytosis. Apoptotic cells were calcein labeled (green) and macrophage nuclei were labeled with DAPI (blue). (**B**) Quantitation of efferocytosis, n = 3. *P < 0.001 NG vs HG + control mimic, **P < 0.01 HG control mimic vs HG + miR126 mimic).

The above data allowed us to speculate that a decrease in p-MERTK and increase in sMER might lead to a decrease in MERTK downstream signaling and impairment of phagocytosis. In an effort to rescue impaired efferocytosis under diabetic conditions, we tested if miR126 overexpression in macrophages reverses impaired engulfment of apoptotic cardiomyocytes (Fig. 6). THP1 cells exposed to NG or HG and transfected with miR-126 mimic or control mimic were overlaid with labeled apoptotic human cardiomyocytes (SV40, green) and engulfment was assessed by microscopy. The number of apoptotic cells ingested by macrophages was reduced in the HG conditions compared to NG (Fig. 6A,B, *P < 0.001). Interestingly, miR-126 mimic transfection significantly increased efferocytosis under high glucose condition as compared to control mimic treated cells (Fig. 6A,B, **P < 0.01).

## Discussion

The microenvironment in diabetes detrimentally affects macrophage function, resulting in adverse remodeling and delay in repair and regeneration^3^. For example, cardiac healing after myocardial ischemia requires accumulation of macrophages at the site of injury for the clearance of apoptotic/dead cardiomyocytes, which is compromised in diabetic conditions^13^,^27^,^28^. Defective efferocytosis in the diabetic condition leads to increased inflammation and necrotic core formation that eventually contributes to delayed wound healing, autoimmune disorders and atherosclerosis^4^,^13^. Prolonged inflammation is also observed in diseases such as cystic fibrosis, asthma, lupus, chronic obstructive pulmonary disease and is also associated with defective efferocytosis^29^. Despite the presence of macrophages, efferocytosis becomes defective implicating a cell-specific insufficiency in clearing the dead cells^1^. Therefore strategies to rescue impaired efferocytosis and related organ failure are clinically relevant.

Although adverse effect of impaired efferocytosis in diabetes mellitus is known, the key players in chronic inflammatory state of diabetic efferocytosis that leads to critical cardiac events are not understood yet. To investigate the molecular mechanisms involved in defective efferocytosis in the HG conditions we targeted miRNAs, considering their emerging role in the cardiovascular pathologies. In this study, we investigated miR-126 role because it is considered to be a potential biomarker of diabetes mellitus (DM) and loss of which, confers an elevated risk of peripheral artery disease, vascular leakage and abnormal angiogenesis^14^,^16^,^17^,^18^. Loss of plasma-miR-126 is considered as type 2 diabetes mellitus-related miRNA signature and is used for early prediction of type 2 DM in susceptible individuals^16^,^18^. MiR-126 regulate gene expression by targeting mRNAs for cleavage or translational repression and both the strands, miR126-3p and miR126-5p have distinctive roles in the cardiovascular system^14^,^30^ with miR126-5p predominantly expressed in pulmonary and cardiac human and mouse tissues^31^. Till date, the role of miR-126 in macrophage efferocytosis (especially in diabetic conditions) has not been explored, although endothelial miR-126 function is widely known. In this study, we found that miR-126 expression is decreased in human diabetic failing heart tissues compared to non-diabetic normal heart tissues, which prompted us to further explore the role of miR-126 in diabetic efferocytosis.

Furthermore, miR-126 plays a critical role in inhibiting breast cancer cell invasion, thyroid cancer cell growth and is used as a therapy for pancreatic cancers^19^,^21^,^32^. In addition, miR-126 has been shown to directly target ADAM9 thereby decreasing the invasiveness of bladder cancer cells^33^. Studies have shown that in cancer cells, miR-126 regulates ADAM9 expression^7^,^21^. To examine the impact of miR-126 on ADAM9, we first overexpressed miR-126 in macrophages using mimics and observed a decrease in ADAM9 expression. In addition, the target validation performed using luciferase assay confirmed that ADAM9 is a direct target of miR-126 in macrophages. ADAM9 expression increases in response to high glucose and this effect could be reversed by transfection of miR126 mimic in macrophages grown under HG conditions. These studies confirm that miR-126 directly targets ADAM9 in macrophages. Progressive increase in ADAM9 expression in cancerous tissue is suggested as a biomarker for poor prognosis in prostate cancer patients^34^,^35^. In the present study, we observed that patients with diabetic failing heart disease have substantial up-regulation of ADAM9 in biopsy samples from the myocardial tissue as compared to non-diabetic normal heart tissues suggesting that ADAM9 may potentially play a key role in cardiac clinical events associated with diabetes.

ADAM family of metalloproteases mediates cellular responses to stress by interacting with several cell surface proteins and regulating processes including ectodomain shedding, proliferation and extracellular matrix binding^36^,^37^. The transmembrane MerTK is shown to be cleaved by ADAM17 leading to generation of soluble form of MerTK (sMer)^12^. The soluble form of MerTK renders the protein inactive and inhibits macrophage clearance of apoptotic cells and platelet aggregation and thrombosis in mice^12^,^22^. Recent studies have shown that MerTK also mediates engulfment of pyrenocytes by central macrophages in erythroblastic islands and promotes the survival of acute lymphoblastic leukemia in the central nervous system referred to as ‘eat me or ‘eat me not’ signals^38^,^39^. MerTK is preferentially expressed by macrophages and thus promotes efferocytosis and inhibits inflammatory response^40^. Since the t-MerTK expression was not altered with HG treatment for 24 hrs, sMer was considered for this study. We observed that sMer is increased in response to HG treatment in macrophages that correlates with the increased ADAM9 expression in these cells. However, the p-MERTK level decreases with HG treatment. Interestingly, ADAMs cleaves MerTK protein under high glucose conditions thus rendering it inactive. Consistent with our *in-vitro* data, the p-MerTK expression was decreased but t-MerTK levels were not altered in the human diabetic failing heart tissues compared with the normal counterparts.

With an aim to rescue the defective efferocytosis, miR-126 was overexpressed using mimics in the macrophages and efferocytosis was assessed. Overexpression of miR-126 increased the percent of efferocytosis in HG conditions in macrophages. These data suggests that miR-126 mimics could possibly be used to rescue the defective efferocytosis in the diabetic patients with heart failure conditions.

In this study, the role of macrophage efferocytosis was considered because macrophages contribute to local homeostatic processes by clearing apoptotic cardiomyocytes^41^. Early after myocardial infarction, the local cardiac and infiltrating macrophages release several factors including cytokines and growth factors with an aim to dissolve and engulf the apoptotic cells, therefore targeting efferocytosis to promote myocardial wound healing might be clinically relevant^41^,^42^,^43^.

**Figure 7:**
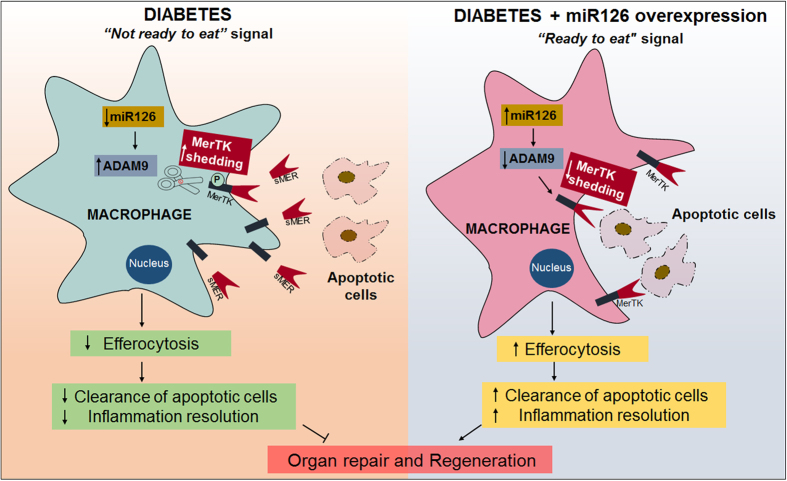
Schematic representation to show the effect of diabetes on efferocytosis. Image on the left shows that diabetes-induced decrease in miR-126 expression results in upregulation of ADAM9 expression that in-turn leads to proteolytic cleavage of MerTK and formation of inactive sMer. The resulting decrease in MerTK phosphorylation (inactivation) leads to reduced downstream cytoskeletal signaling required for engulfment and thus decreases efferocytosis of apoptotic cells and lower inflammation resolution, which eventually results in defective organ repair. This is termed as “not ready to eat” signal of macrophages in the diabetic conditions. Image on the right depicts that overexpression of miR-126 suppresses ADAM9 expression, which in turn rescues efferocytosis in diabetic conditions. This sends “ready to eat signal” from the miR-126 overexpressed macrophages in diabetic conditions. This eventually results in improved organ repair and regeneration. This we termed as “ready to eat” signal from the macrophages.

In summary (Fig. 7), we describe a novel cellular pathway involved in diabetic efferocytosis, wherein diabetes-induced decrease in miR-126 expression results in upregulation of ADAM9 expression that in-turn leads proteolytic cleavage of MerTK and formation of inactive sMer. The resulting decrease in MerTK phosphorylation (inactivation) leads to reduced downstream cytoskeletal signaling required for engulfment and thus decreases efferocytosis. This is termed as “not ready to eat” signal of macrophages in the diabetic conditions. We propose that overexpression of miR-126 suppresses ADAM9 expression, which in turn rescues efferocytosis in diabetic conditions. This sends “ready to eat signal” from the miR-126 overexpressed macrophages in diabetic conditions. This pathway could be intervened at multiple levels for example, miR-126 mimics could be administered, ADAM9 expression could be targeted or the downstream MerTK could be addressed for therapeutic purpose. Thus, understanding of the mechanisms involved in macrophage-induced efferocytic clearance of apoptotic cells might lead to novel therapeutic strategies directed for the related-organ regeneration after injury.

## Methods

### Cell culture and reagents

RAW 264.7 cells (mouse mononuclear/macrophage cell line, ATCC) were cultured in DMEM (Life technologies, Grand Island, NY) with 10% FCS (ATCC, Manassas, VA), and 1% Penicillin-Streptomycin (HyClone). Bone marrow-derived macrophages (BMM) were isolated from the bone marrow of non-diabetic control (wild type) and diabetic (db/db) mice as described in our previous studies^44^,^45^. In brief, BMM was isolated from mice by density-gradient centrifugation with Histopaque-1083 (Sigma). Macrophages were selected by allowing attachment to cell-culture plate for 1 hour and the unattached cells were removed. BMM were cultured in DMEM macrophage media (Life technologies, Grand Island, NY). RAW 264.7 cells were cultured in DMEM medium under normoglycemia (NG; 5 mM) or hyperglycemia (HG; 35 mM) conditions or osmotic control (5 mM glucose + 30 mM Mannitol). ADAM9 mRNA and protein expression was blocked as described in the previous publications^25^,^26^ using small molecules, genistein (10 μM) and ebselen (20 μM), respectively.

### Transfections

MiR-126 mimic, miR-126 inhibitor and their respective nonspecific control (mirVana^TM^miRNA mimic negative control, Ambion, Thermo Fisher Scientific, U.S.A) were used for transfection as described previously^44^. Briefly, RAW 264.7 cells were seeded in 6-well plates and subjected to NG or HG conditions 48 hours prior to transfection. The miR-126 mimic, inhibitor and the respective controls were added at the final concentration of 60 nM with Lipofectamine Transfection Reagent (Invitrogen, Thermo Fisher Scientific, USA) according to the manufacturer’s instructions. After 48 hours, the cells from each group were either used for efferocytosis assay or harvested, for PCR, immunoprecipitation and Western blot analysis.

### Luciferase assay

Macrophages (RAW 264.7 cells) were co-transfected with miR-126 mimic (60 nm/l), or miR mimic control (60 nm/l) and a reporter plasmid containing the 3′untranslated region (3′-UTR) of ADAM9 (ADAM9; MmiT077372-MT06, 100 ng, GeneCopoeia, Rockville, MD) or corresponding control empty luciferase reporter vector (CmiT000001-MT06, 100 ng, GeneCopoeia, Rockville, MD). The miR and reporter plasmid were mixed with Lipofectamine 2000 (Thermo Fisher Scientific, Tewksbury, Massachusetts) and added to a 48-well plate containing RAW 264.7 cells (1.5 × 10^4^). After a 24-h transfection, luciferase activity was assessed using a dual luciferase reporter assay kit (Promega, Madison, Wisconsin) per manufacturer’s protocol.

### Efferocytosis assay

Efferocytosis assay was performed as described previously at a 1:1 ratio of macrophage: apoptotic cells (ACs)^23^. The ratio was determined in a pilot study under hyperglycemia conditions. RAW 264.7 macrophages were plated at 5 × 10^4^ cells/well in 8-well glass slides followed by treatment with NG and HG conditions. Immortalized human ventricular cardiomyocytes- SV40 (5 × 10^4^ cells/well, Applied Biological Materials Inc, Richmond, Canada) or C2C12 (mouse myoblasts, 5 × 10^4^ cells/well, ATCC, Manassas, VA) were grown according to the manufacturer’s instructions. The cells were labeled with Calcein AM (Thermo Fisher Scientific) for 2 h followed by wash with PBS. Apoptosis in C2C12 or SV40 cells was induced by UV exposure (Spectroline, Westbury, NY) for 10 minutes. The apoptotic cells (ACs) were overlaid on the RAW 264.7 cells or bone marrow-derived macrophages and incubated for 2 hours. Following efferocytosis, cells were washed 4 times with ice-cold PBS to remove the ACs that were not engulfed. The cells were fixed with 1% paraformaldehyde, DAPI stained (Thermo Fisher Scientific) and mounted for fluorescent microscopy (EVOS FL, Thermo Fisher Scientific) and confocal analysis (Nikon A1 Confocal Imaging System). Efferocytosis was determined by counting cells containing engulfed green fluorescent apoptotic bodies. A minimum of 100 macrophages was counted per well in triplicate. Data was represented as percent (%) efferocytosis- total number of cells with ingested apoptotic cells divided by the total number of macrophages counted times 100. As an additional control for efferocytosis, cytochalasin (2 μM) was used to disrupt the cell membrane that inhibits efferocytosis. The protocol is explained in detail in Fig. S2. Flow cytometry was performed for analyzing the engulfment of the dead cells using Cell Analyzers (BD LSRII & BD FACS Fortessa). For flow cytometry analysis, pHrodo green dye^46^, which is a non-fluorescent dye at neutral pH was used to label the dead cells, upon internalization the acidic environment in the macrophages elicits a bright green fluroscence indicating the ingestion of dead cells as compared to the control DAPI-viable cells (Fig. S2).

### Human heart tissue analysis

Heart tissue samples were obtained from failing human hearts at the time of cardiac transplantation at the Houston Methodist DeBakey Heart & Vascular Center, Houston Methodist Hospital (Houston, Texas). The tissues were immediately frozen in liquid nitrogen and stored at −80 °C until use. Normal tissue samples were obtained from donor hearts that were not used for transplantation and were collected and stored in the same manner. Informed consent was obtained from all subjects. All tissues were collected under an approved Houston Methodist Research Institutional Review Board protocol. Experiments involving human tissue samples were performed in accordance with approved guidelines and regulations by the Houston Methodist Research Institutional Review Board.

### Real time PCR

Total RNA was extracted from cells or tissues using a RNA extraction kit (Qiagen) according to the manufacturers’ instructions. RNA was reverse transcribed to cDNA using Q-script kit (Quanta Biosciences, Gaithersburg, MD) and TaqMan MiRNA Reverse Transcription Kit (Applied Biosystems, Foster City, CA) according to manufacturer’s instructions. Quantitative real-time PCR was performed in a Quanta bio-studio (Applied Biosystems, Grand Island, NY) using the Taqman–based method for detection of gene amplification according to the manufacturer’s instructions. Relative miRNA or mRNA expression of target gene was normalized to the U6 control or GAPDH gene, respectively. Data was represented as fold change versus respective control.

### Immunohistochemistry

Immunohistochemistry was performed as described previously^47^. In brief, paraffin-embedded tissue sections (5 μm) were deparaffinized, hydrated and washed in TBS (10 mM/l Tris HCl, 0.85% NaCl, and pH 7.5) containing 0.1% bovine serum albumin. Endogenous peroxidase activity was quenched by incubating the slides in methanol and 0.6% H_2_O_2_ in methanol. In all cases, the primary antibody, namely MERTK (1:100), phospho-MERTK (1:50) and ADAM9 (1:50) (Abcam, Cambridge, MA), was incubated overnight at 4 °C. The slides were washed in TBS, and HRP-conjugated secondary antibody (Santa Cruz Bio-technology, Dallas, Texas) was added and the sections were further incubated at room temperature for 45 min. The sections were washed in TBS and incubated with diaminobenzidine tetrahydrochloride as the substrate, and counterstained with hematoxylin and observed under light microscope.

### Protein isolation and Immunoprecipitation

Protein isolation for cultured cells were performed as previously described^44^,^48^. Briefly, cells were homogenized in lysis buffer (Cell Signaling Technology, MA, USA) containing 20 mmol/l Tris-HCl [pH 7.5], 150 mmol/l NaCl, 2.5 mmol/l sodium pyrophosphate, 1 mmol/l β-glycerophosphate, 1 mmol/l sodium orthovanadate, 1 μg/ml leupeptin, 1 mmol/l ethylenediaminetetraacetic acid [EDTA], 1 mmol/l ethylene glycol tetraacetic acid [EGTA], 1% Triton X-100, protease and phosphatase inhibitors (Thermo Fisher Scientific). Immunoprecipitation was performed by incubating cell lysates with 1 μg of MERTK antibody targeted to the intracellular C-terminus domain (Abcam, Cat # ab95925) or IgG control antibody for overnight (12 h) with gentle rotation. Similarly, the conditioned media from the macrophage culture was incubated with MERTK antibody targeted to extracellular domain (R&D systems, Minneapolis, MN; Cat # AF591). Subsequently, protein G-Sepharose beads (Invitrogen, South San Francisco, CA) were added and complexes rocked for an additional 4 hours at 4 °C. Beads were collected by centrifugation and washed 4 times with cold PBS. Bound proteins were eluted in 2X SDS buffer and subjected to SDS-PAGE.

### Western blot analysis

Equal amounts of protein were separated by 10% SDS-PAGE and blotted onto polyvinylidenedifluoride (PVDF) membranes (Bio-Rad, Hercules, CA). The blots were incubated with antibodies against phosho-MERTK antibody (1:500, Abcam, Cat# ab192649), total MERTK (LS bioscience, Cat# LS-C99094), GAPDH (Cell Signaling) and developed with an enhanced chemiluminescence detection system (Amersham, Piscataway, NJ). Densitometry of the bands from the western blot was analysed using ImageJ software.

### Statistical Analysis

Analyses were performed using Prism 6 (GraphPad Software, La Jolla, CA, USA) using non-parametric statistics. Mann–Whitney’s tests were used to compare between two groups. When more than two groups were involved, analysis of variance with a Bonferroni multiple comparison test was used to analyze the data. *P*-values less than 0.05 were considered to be statistically significant.

## Additional Information

**How to cite this article**: Suresh Babu. S. *et al*. MicroRNA-126 overexpression rescues diabetes-induced impairment in efferocytosis of apoptotic cardiomyocytes. *Sci. Rep.* **6**, 36207; doi: 10.1038/srep36207 (2016).

**Publisher’s note:** Springer Nature remains neutral with regard to jurisdictional claims in published maps and institutional affiliations.

## Supplementary Material

Supplementary Information
