# Supplementary material for: MicroRNA-126 overexpression rescues diabetes-induced impairment in efferocytosis of apoptotic cardiomyocytes
Source: Sci Rep. 2016 Nov 9;6:36207. doi: 10.1038/srep36207 (PMC5101812; doi:10.1038/srep36207)
Supplement: Supplementary Information [file srep36207-s1.pdf]

Supplementary info

MicroRNA-126 overexpression rescues diabetes-induced impairment in efferocytosis of apoptotic cardiomyocytes

Sahana Suresh Babu†, Rajarajan A. Thandavarayan†, Darukeshwara Joladarashi$, Prince Jeyabal†, Shashirekha Krishnamurthy†, Arvind Bhimaraj§, Keith A. Youker§, Prasanna Krishnamurthy *$†

† Department of Cardiovascular Sciences, Centre for Cardiovascular Regeneration, Houston Methodist Research Institute, Houston, TX 77030, USA

§ Houston Methodist DeBakey Heart & Vascular Center, Houston Methodist Hospital, Houston, Texas, USA

$ Department of Biomedical Engineering, University of Alabama at Birmingham, AL 35294, USA

Figure S1

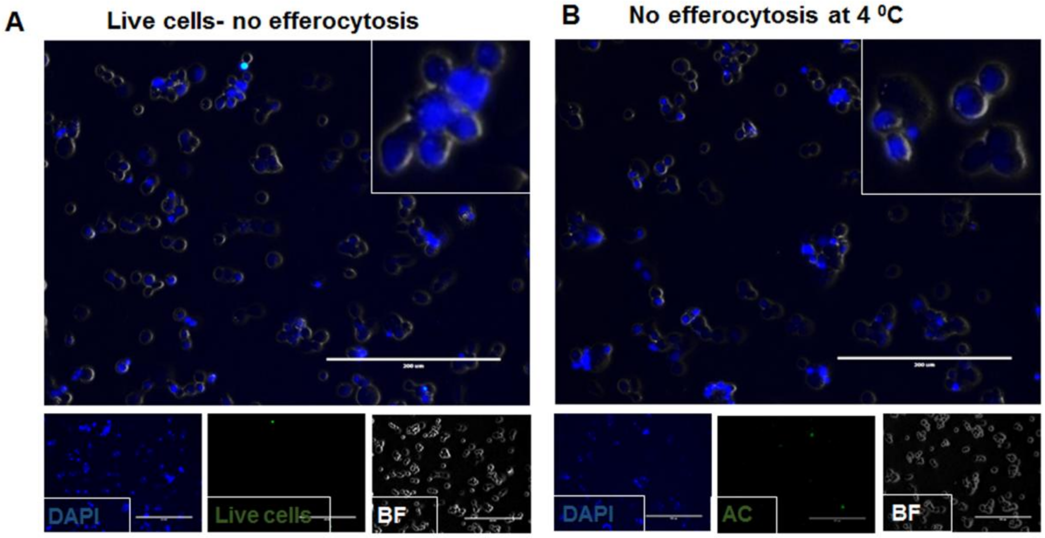

Figure S1: Fluorescent microscopy- efferocytosis assay under different experimental conditions. (A) Lack of engulfment of (calcein labeled green) live human ventricular cardiomyocytes by RAW 264.7 cells incubated at 37°C. (B) Inhibition of efferocytosis of apoptotic cells (green) by RAW 264.7 cells at 4°C. Macrophage nuclei is DAPI stained (blue).

Fig S2

1. **Preparation of apoptotic cells**
2. **Transfect RAW 264.7 cells with miR126-mimic or control mimic**
3. **Overlay apoptotic C2C12 cells on RAW 264.7 cells. Efferocytosis at 37°C. Cytochalasin D (2μM) disrupts cell membrane, hence no efferocytosis (no engulfment of green cells)**
4. **Flow cytometry**
5. **Confocal Microscopy**

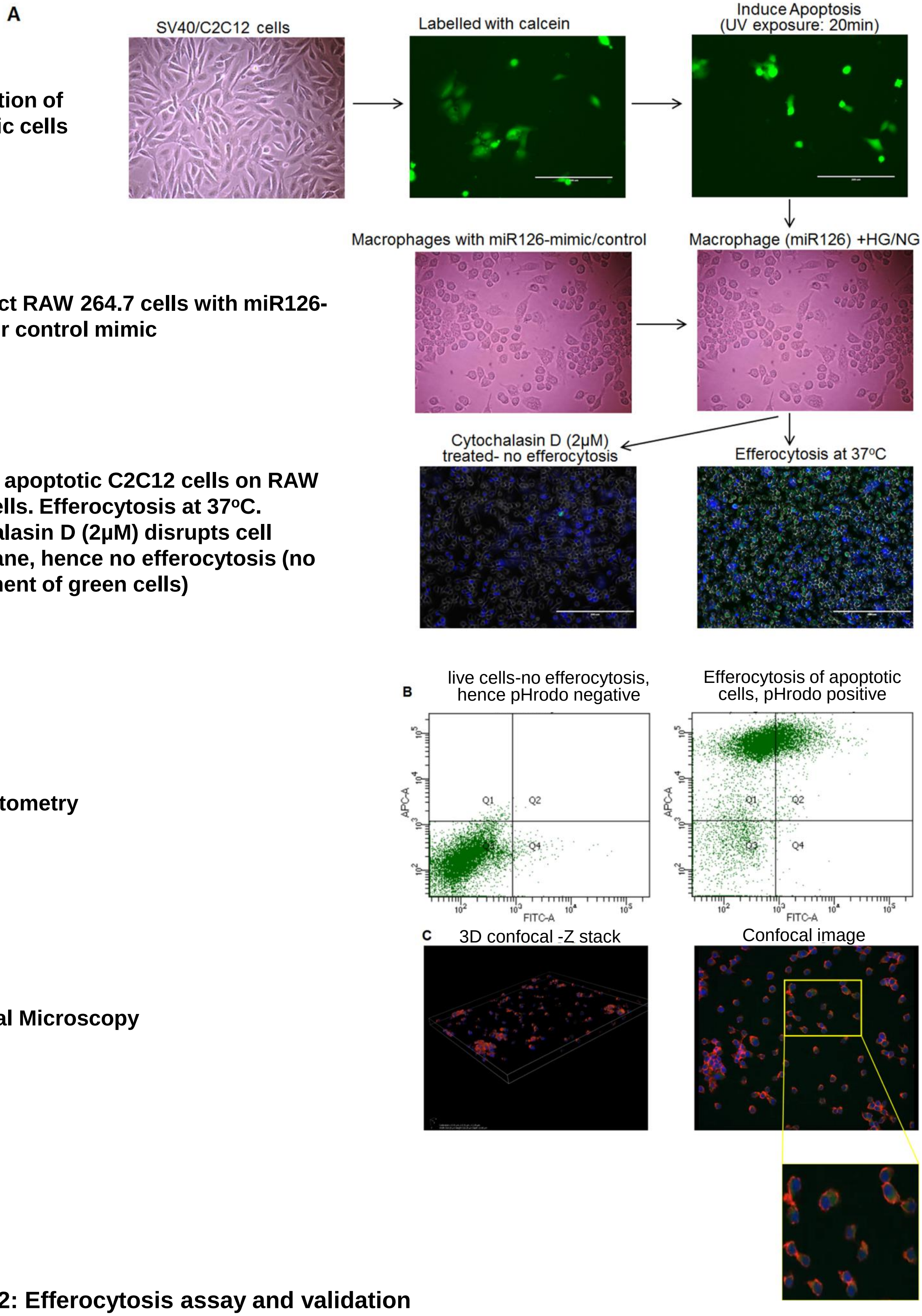

Figure S2: Efferocytosis assay and validation

(A) Efferocytosis assay. Pre-incubation of macrophages with cytochalasin D (2μM, to disrupt actin polymerization) inhibits efferocytosis and did not show engulfment of the apoptotic cells. (B) Flow cytometry analysis, engulfment of phRodo green labeled apoptotic cells by macrophages (stained with anti-CD11b-APC). Macrophages exhibit increasing fluorescence (green) due to acidification of endocytic compartments in comparison to the dye being nonfluorescent at neutral pH (or lack of efferocytosis of live cells). Representative z-stack image from 3D confocal imaging of corresponding video of phagocytic cells (Red: F-actin for macrophages, Green: calcein labeled apoptotic cells, blue nuclei: DAPI).

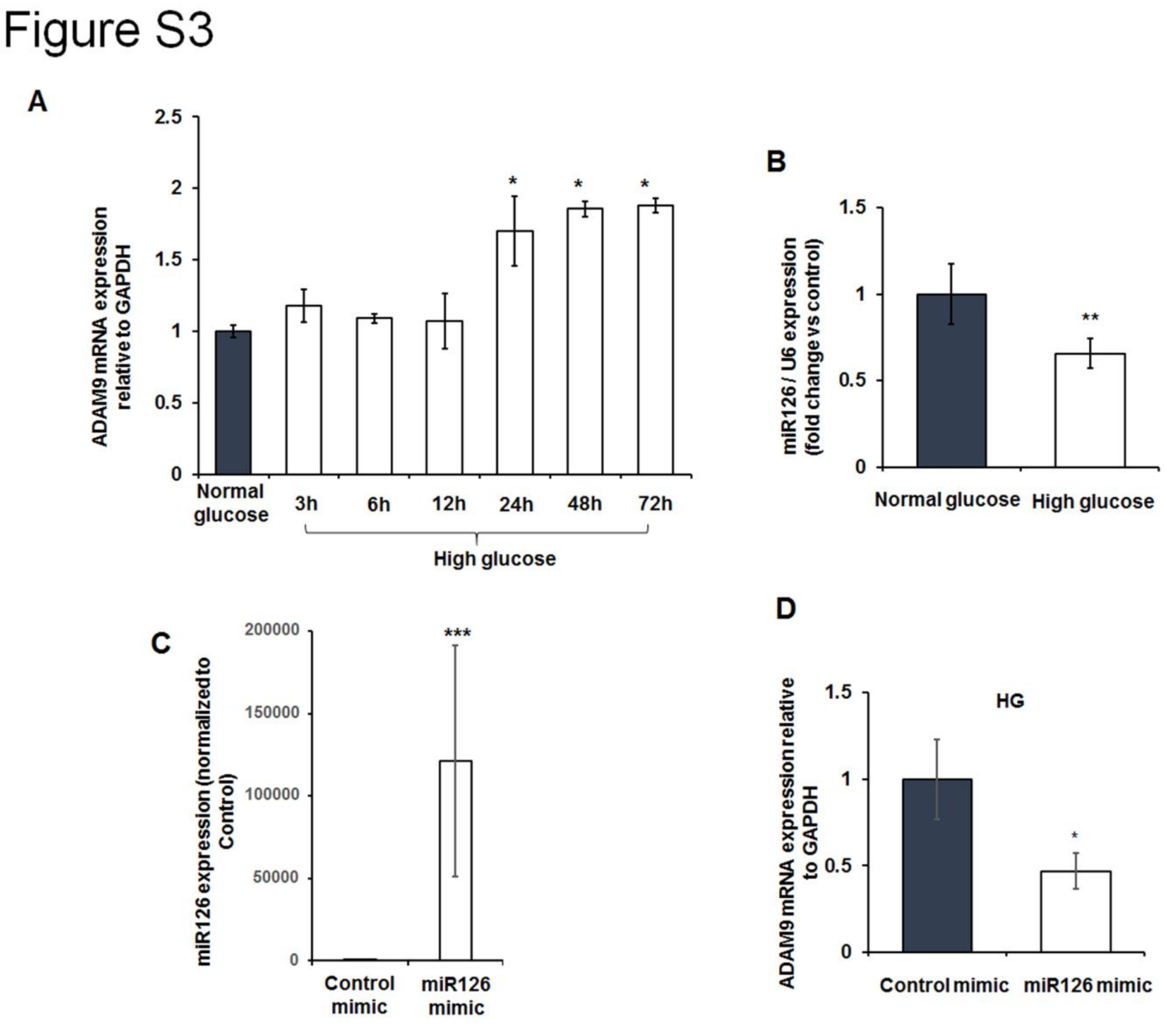

Figure S3: ADAM9 expression and miR-126 expression in RAW 264.7 cells.
(A) miR-126 expression measured in the RAW 264.7 cells subjected to normoglycemia (NG) and high glucose (HG) conditions (Normalized to U6, n = 5, **P < 0.01). (B) miR-126 mimic transfection in RAW 264.7 cells increases miR-126 expression as compared to control mimic transfections (normalized to U6, n = 5, ***P < 0.001). (C) ADAM9 mRNA expression in RAW cells subjected to NG and HG conditions at indicated time point (normalized to GAPDH, n = 3, *P < 0.05). (D) Under high glucose condition, miR-126 mimic treated RAW 264.7 cells show decrease in ADAM9 mRNA levels compared to control mimic transfected cells (n = 3. *P < 0.05, normalized to control GAPDH).

Figure S4

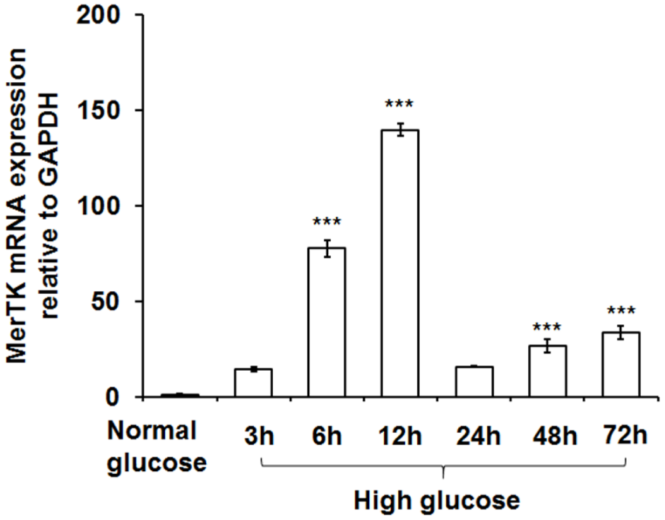

Figure S4: MerTK expression in RAW 264.7cells exposed to high glucose.
MerTK mRNA expression in RAW 264.7 cells subjected to NG and HG conditions at indicated time points (normalized to GAPDH, n = 3, ***P < 0.05).
